# Stability of infliximab serum concentrations during long-term frozen storage: a paired biobank study

**DOI:** 10.1093/ecco-jcc/jjag113

**Published:** 2026-07-31

**Authors:** Suzanne I Anjie, Sarah van Zon, Karien Bloem, Theo Rispens, Geert R D’Haens

**Affiliations:** Department of Gastroenterology and Hepatology, Amsterdam UMC, Amsterdam, The Netherlands; Department of Gastroenterology and Hepatology, Amsterdam UMC, Amsterdam, The Netherlands; PurSang Diagnostiek, Biologics and Immunopathology Lab, Amsterdam, The Netherlands; Sanquin Research, Amsterdam, The Netherlands; Amsterdam Institute for Infection and Immunity, Amsterdam UMC, Amsterdam, The Netherlands; Department of Molecular Cell Biology and Immunology, Amsterdam UMC, Vrije Universiteit Amsterdam, Amsterdam, The Netherlands; Department of Gastroenterology and Hepatology, Amsterdam UMC, Amsterdam, The Netherlands

**Keywords:** infliximab, therapeutic drug monitoring, biobanking, sample stability

## Abstract

**Background:**

Pharmacokinetic research of infliximab often relies on prospectively collected, long-term frozen serum samples. Although the effect of storage conditions on infliximab stability has been researched extensively, the influence of *long-term storage* on the reliability of infliximab concentration measurements remains unknown.

**Methods:**

In this proof-of-concept study, infliximab serum concentrations measured after 2, 5, and 8 years of standardized frozen storage at −70 °C were compared with paired samples analyzed directly after collection. Equivalence was assessed using a two one-sided tests framework with predefined margins of 80%-125%, in line with FDA equivalence guidance.

**Results:**

Equivalence was demonstrated at all three storage durations. Geometric mean ratios were 87.0% (90% CI, 80.9%-93.6%), 103.2% (90% CI, 90.3%-117.9%), and 99.3% (90% CI, 92.5%-106.6%) at 2, 5, and 8 years, respectively, all within predefined equivalence margins. A statistically significant reduction was observed in the 2-year group (*P* = .010), though within equivalence bounds. No concentration-dependent bias was detected (*P* = .926), and Spearman correlation between fresh and frozen concentrations was high (*ρ* = 0.908, *P* < .001).

**Conclusion:**

Infliximab serum concentrations remain stable after long-term frozen storage of up to 8 years under standardized conditions, supporting the validity of deferred concentration analyses in prospectively stored serum samples.

## 1. Introduction

Monoclonal antibodies, including infliximab, are widely used in the treatment of patients with inflammatory bowel disease (IBD).^1^ In clinical practice, infliximab concentrations are typically measured shortly after sample collection, with results available within days. However, in research contexts, serum is usually stored in clinical biobanks or as part of prospective cohort studies, with concentration analyses performed months to even many years later. This storage time often exceeds the time normally included as the stability parameter in the validation of the drug concentration assay.^2^ Ensuring the stability of infliximab following long-term storage is critical for the validity and interpretability.

However, long-time storage introduces several potential sources of variability beyond biological degradation of the monoclonal antibody itself: changes in assay reagents or analytical platforms over time, and pre-analytical factors such storage conditions.^3^ In addition, previous studies have shown that even limited freeze–thaw exposure may decrease infliximab stability in a sodium chloride buffer, caused by increased aggregation and alterations in physicochemical characteristics.^4^ However, the influence of storage duration, rather than storage conditions on infliximab concentrations in serum has not yet been studied. The expanding pharmacokinetic research in IBD makes empirical validation of long-term sample stability essential. In this study, we systematically investigated the infliximab serum concentration stability following long-term storage under controlled conditions.

## 2. Methods

In this proof-of-concept study, the infliximab serum concentration stability was measured using a paired study design. Serum samples from infliximab-treated IBD patients were collected at a single time point and aliquoted into two vials: one analyzed in standard of care and one stored long-term in our fully standardized and quality-controlled biobank. Patients in the induction phase of treatment or with measurable anti-drug antibodies were excluded.

Blood samples were collected in serum separator tubes and allowed to coagulate for at least 30 minutes at room temperature before centrifugation at 2000 × g (3000 rpm) for 10 minutes at room temperature. Serum was collected and aliquoted into two 0.5 mL microtubes. One microtube was analyzed directly, and one microtube was kept at −70 °C until analysis. The infliximab concentration was analyzed using a validated ELISA assay (ISO 15189 accredited) as described previously.^5^

### 2.1 Statistical analysis

Sample size was calculated using a two one-sided tests (TOST) equivalence framework, with a significance level of α = 0.05, power of 80%, expected difference of 0%, common standard deviation of 10%, and equivalence margins of −20% to + 25%.^2^ This yielded a required sample size of *n* = 5 per storage duration group. With *n* = 7 samples per group, the achieved power was 97%.

For each paired sample, the ratio of frozen to fresh infliximab concentration was calculated and log-transformed (ln[frozen/fresh]) prior to analysis, because biologic drug concentrations follow a log-normal distribution and render the equivalence margins symmetric: the predefined margins correspond to ± ln(1.25) = ±0.2231 on the log scale. Equivalence was assessed per storage duration group using TOST at α = 0.05 on the log-transformed ratios, with equivalence declared when the 90% confidence interval (CI) of the mean log-ratio fell entirely within [−0.2231, + 0.2231]. Results were back-transformed and reported as geometric mean ratios (GMR) with 90% CIs. To assess overall agreement between fresh and frozen measurements, Spearman’s rank correlation and 95% CI were calculated across all paired samples and per time point. Linear regression of frozen on fresh concentrations was performed to assess concordance. To exclude concentration-dependent bias, a separate linear regression was performed with the ln-transformed ratio as the dependent variable and the mean of fresh and frozen concentrations as the independent variable. Agreement was further evaluated using a Bland-Altman analysis with ratio (frozen/fresh) on the *Y*-axis; limits of agreement (LoA) were calculated on the log scale as exp(mean ln-ratio ± 1.96 × SD ln-ratio) and back-transformed. Cohen’s kappa coefficient was used to assess agreement when infliximab concentrations were categorized as subtherapeutic (<5 µg/mL), therapeutic (5-10 µg/mL), or supratherapeutic (>10 µg/mL). All analyses were performed using SPSS Statistics version 28.0 or higher (IBM Corp., Armonk, NY, USA) and GraphPad Prism (version 10.6.0; Graphpad Software, San Diego, CA, USA).

## 3. Results

A total of 21 IBD patients with paired samples available were included (*n* = 5 ulcerative colitis; *n* = 16 Crohn’s disease). Fresh infliximab concentrations ranged from 0.4 to 12.6 mg/L across all samples. Storage time of the paired sample was either 2 years (23.96 months ± 1.55), 5 years (60.43 months ± 2.79), or 8 years (96.0 months ± 1.5). All patients were in the maintenance phase of infliximab treatment, with a median treatment period of 23.1 weeks (IQR 16.9-41.0).

### 3.1 Equivalence analysis

**Figure 1.**
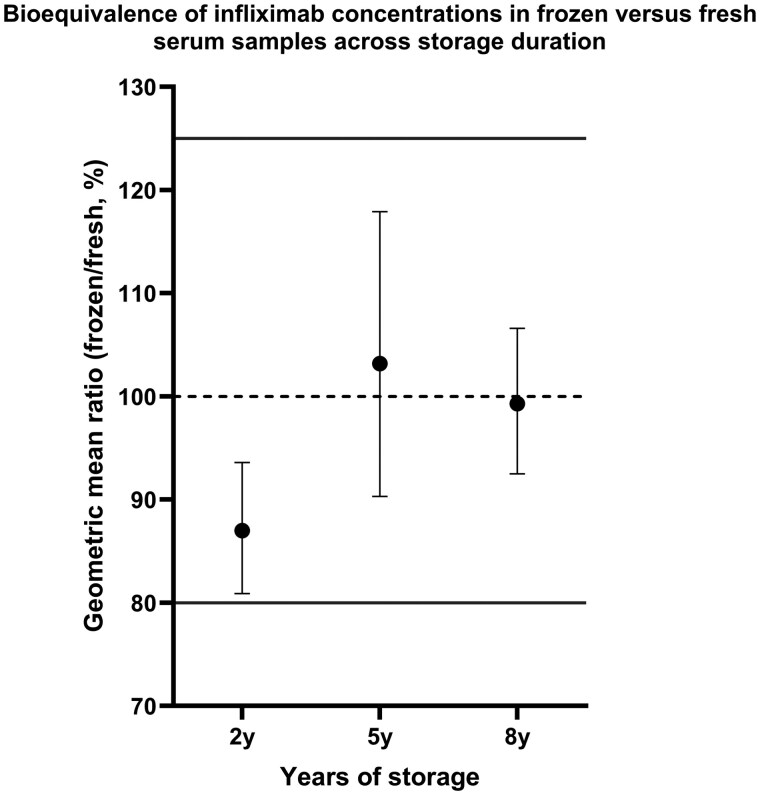
Geometric mean ratios (GMR) of frozen to fresh infliximab concentrations per storage duration group. GMR with 90% (CI are shown for samples stored for 2, 5, and 8 years (*n* = 7 per group). The dashed vertical lines represent the predefined equivalence margins of 80%-125%. Equivalence is demonstrated when the 90% CI falls entirely within these margins. Abbreviations: GMR, geometric mean ratios; CI, confidence intervals.

Equivalence between fresh and frozen infliximab concentrations was demonstrated at all three storage durations. The GMR was 87.0% (90% CI, 80.9%-93.6%) at 2 years, 103.2% (90% CI, 90.3%-117.9%) at 5 years, and 99.3% (90% CI, 92.5%-106.6%) at 8 years, all 90% CIs fell within the predefined equivalence margins of 80%-125% (Figure 1). A statistically significant reduction in frozen vs fresh concentrations was observed in the 2-year group (mean ln-ratio −0.139, SD 0.099; *P* = .010), though the GMR remained within equivalence bounds. No significant difference was observed at 5 years (mean ln-ratio + 0.031, SD 0.182; *P* = .663) or 8 years (mean ln-ratio −0.007, SD 0.096; *P* = .850). Across all 21 paired samples, the overall GMR was 96.2% (95% CI, 90.1%-102.8%), with a mean ln-ratio of −0.038 (SD 0.146), which did not significantly differ from zero (t(20) = −1.206, *P* = .242).

### 3.2 Agreement and bias analyses

**Figure 2.**
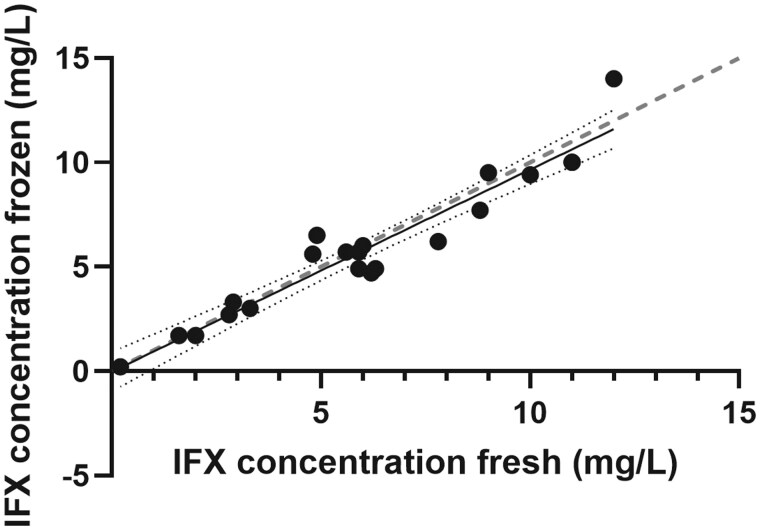
Concordance between fresh and frozen infliximab concentrations. Scatter plot of frozen vs fresh infliximab concentrations (mg/L) for all 21 paired samples, displayed on a log10 scale. The dashed line represents perfect concordance (identity line, slope = 1, intercept = 0). The solid line represents the linear regression fit (slope = 0.968, 95% CI, 0.831-1.105; *y*-intercept = −0.022, 95% CI, −0.968 to + 0.923; *R*^2^ = 0.920, *P* < .001). Spearman correlation coefficient for all samples: ρ = 0.908, *P* < .001.

**Figure 3.**
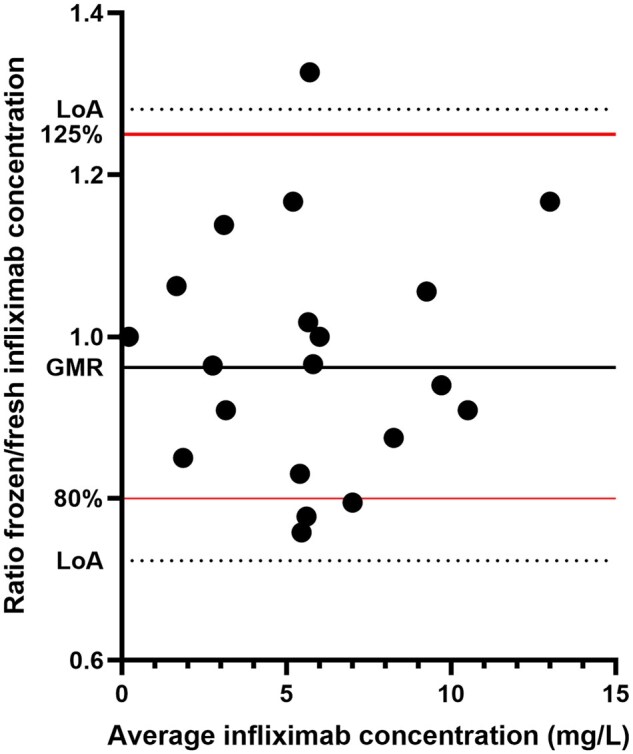
Bland-Altman plot of frozen to fresh infliximab concentration ratios. The ratio of frozen to fresh infliximab concentration (frozen/fresh) is plotted against the mean of the two measurements for all 21 paired samples. The solid black line represents the GMR (GMR = 0.962, mean bias −3.8%). Dashed gray lines indicate the LoA (0.723-1.281), calculated on the log scale as exp(mean ln-ratio ± 1.96 × SD ln-ratio) and back-transformed. Red lines indicate the predefined equivalence margins of 80% and 125%. Abbreviations: GMR, geometric mean ratios; LoA, limits of agreement.

Spearman correlation between fresh and frozen concentrations was high across all samples (*ρ* = 0.908, 95% CI, 0.900-0.962, *P* < .001), and remained significant within each storage group (2 years: *ρ* = 0.755, 95% CI, 0.003-0.961, *P* = .050; 5 years: *ρ* = 0.929, 95% CI, 0.584-0.990, *P* = .003; 8 years: *ρ* = 0.991, 95% CI, 0.938-0.999, *P* < .001; Figure 2). Linear regression of frozen on fresh concentrations demonstrated a slope of 0.968 (95% CI, 0.831-1.105), not significantly different from unity, and a *y*-intercept of −0.022 (95% CI, −0.968 to + 0.923), not significantly different from zero (*R*^2^ = 0.920). Bland-Altman analysis showed a GMR of 0.962 (mean bias −3.8%) with LoA of 0.723-1.281 (Figure 3). One sample exceeded the upper equivalence bound (LoA upper limit 1.281 vs equivalence margin 1.25). Linear regression of the ln-ratio against mean concentration showed no significant association (*B* = 0.001, 95% CI, −0.020 to 0.022, *R*^2^ < 0.001, *P* = .926), confirming the absence of concentration-dependent bias. Categorizing infliximab concentrations as subtherapeutic (<5 μg/mL), therapeutic (5-10 μg/mL), or supratherapeutic (>10 μg/mL), agreement between fresh and frozen measurements was moderate (Cohen’s κ = 0.426, *P* = .014), with 66.7% of samples (14/21) classified into the same category. Discordant classifications occurred exclusively between adjacent categories.

## 4. Discussion

In this proof-of-concept study, we demonstrated that infliximab serum concentrations remain stable after long-term frozen storage of up to 8 years. Equivalence between fresh and frozen measurements was confirmed at all three storage durations, with GMRs ranging from 87.0% to 103.2%, all within the predefined equivalence margins.

As pharmacokinetic research often relies on long-term stored serum, the stability of the infliximab serum concentration needed to be verified. Our findings provide direct support for the validity of this approach within the concentration range and storage conditions studied and are directly relevant to the use of frozen samples.

A statistically significant reduction in frozen vs fresh concentrations was observed in the 2-year storage group. Although this reduction remained within the predefined equivalence margins, it was the only group to demonstrate a systematic directional shift. This pattern may reflect early-phase protein instability, a counterintuitive finding given that degradation would be expected to accumulate over time. An alternative explanation is variability in sample handling or due to assay variation over time. This could have resulted in the slightly higher reported infliximab concentration at the time of the fresh measurement for the 2-year group, rather than biological degradation. Assay performance is carefully monitored over time, eg, by the inclusion of a quality control sample and assay monitoring using Westgard rules. In addition, according to ISO15189 regulations, yearly checks are performed, and the assay is included in external quality assessments. However, due to the nature of the assays, small variations can be present. As no power calculation was performed for this subgroup analysis, this finding should be interpreted with caution.

Beyond group-level equivalence, the agreement analyses collectively support the analytical reliability and long-term assay performance of frozen samples. The absence of concentration-dependent bias indicates that the assay performs consistently regardless of drug level, which is particularly relevant given the wide concentration range encountered in clinical practice. The marginal excess of the upper LoA beyond the equivalence bound is consistent with the exploratory sample size and does not alter the overall conclusion but does highlight that individual-level interchangeability cannot be guaranteed in all cases: a nuance worth acknowledging when applying these findings to single-patient clinical decisions. This is further illustrated by the moderate agreement observed when concentrations were categorized into clinically relevant thresholds.

This study has several limitations. The sample size of seven per group, while adequately powered for the primary equivalence analysis, limits the precision of subgroup estimates. In the 2-year group in particular, the 90% CI approached the lower equivalence margin. Furthermore, only samples without detectable anti-drug antibodies were included. Given that immunogenicity may independently affect measurable drug concentrations, the stability of frozen infliximab samples containing antibody-drug complexes remains to be established. Moreover, our study only investigated the stability of infliximab. Although differences for other monoclonal antibodies might not be expected from a biochemical perspective, extrapolation of the findings of this study to the stability of other biologic agents requires further investigation. Strengths include the paired design, which minimizes inter-individual variability, and the use of a well-characterized biobank with standardized storage conditions.

In conclusion, infliximab serum concentrations are stable after long-term frozen storage of up to 8 years, supporting the validity of deferred concentration analyses in prospectively collected frozen serum samples used for clinical research.

## Data Availability

All data relevant to the study are included in the article or uploaded as supplementary information. Individual participant data are not publicly available due to privacy and ethical restrictions, as these data cannot be fully de-identified. Further information may be available upon reasonable request and subject to review by the appropriate ethics committee and data protection regulations.
